# The Planned Alternating Pathways: a flexible protocol for working with anorexic and bulimic adolescents and their families

**DOI:** 10.3389/fpsyg.2025.1603246

**Published:** 2025-05-19

**Authors:** Valeria Ugazio, Lisa Chiara Fellin

**Affiliations:** ^1^European Institute of Systemic-relational Therapies, Milan, Italy; ^2^Department of Human and Social Sciences, University of Bergamo, Bergamo, Italy

**Keywords:** eating disorders, anorexia/bulimia, therapeutic alliance in adolescence, systemic therapies, family therapeutic alliance, adolescent eating disorders, semantic polarities, family semantic polarity theory

## Abstract

The article presents the Planned Alternating Pathways, a flexible systemic protocol structured into four phases for working with adolescent with anorexia and bulimia and their families. Largely inspired by the Family Semantic Polarity Theory, it maintains the assumption of pioneering systemic approaches that anorexia and bulimia are coping strategies, albeit very dangerous. The protocol aims to empower the family as a whole. Its main purpose is to change the positions of the patient and the family members who feel and/or are considered to be at a disadvantage, while reducing internal competition and polarization between so-called “winners” and “losers.” These are particularly harsh in families with eating disorders, where the semantic of power generally prevails. The protocol places tailored psychotherapy at its centre and revolves around three main objectives: (1) to maximise the generally fragile therapeutic alliance with the family and above all with the patient; (2) to lengthen the treatment; 3) to overcome a typical therapeutic dilemma of eating disorders concerning the duration of the treatment. The psychotherapeutic process should be long enough to address the identity issues of the patient with anorexia or bulimia, but as soon as the patient regains a normal or near-normal weight, parents drop out therapy or put the therapeutic team in the position of terminating it. The introduction of an individual phase helps to prevent this too early conclusion of treatment and distinguishes the format from that of traditional family therapy. The individual path approaches the patient’s emotions, feelings and moods in an indirect way, while helping the patient to discover her hidden talent, often suppressed by “competitive trails.” This phase also paves the way for a possible return to therapy, should existential issues arise in the future. The protocol can be extended to the treatment of adolescents or young adults with other problems and disorders, because it takes into account the changes that have occurred in all families that make it difficult long-term involvement of the whole family in the therapeutic experience. It also enhances the family’s resources while reduces the asymmetry between patients and therapists increasingly unpalatable in Western societies.

## 1 A quest for new approaches

There is at least one point of unanimous consensus in the extensive literature on eating disorders: the treatment outcomes are modest and discouraging. This calls for new clinical and research perspectives (Grilo, 2024; Marzola et al., 2021, 2022; Monteleone and Cascino, 2021; Toppino et al., 2024; Touyz et al., 2023).

A recent example is an article (Touyz et al., 2023), authored by some of the most influential figures in the field, in which the authors revisit the question: “What kind of illness is anorexia?”— already raised two decades before (Beumont and Touyz, 2003). They argue that reflecting on such a fundamental question is necessary, given the disappointing outcomes: only 30% of surviving anorexia patients have fully recovered ten years after onset of symptoms. Moreover, recovery rates are often overestimated (Södersten et al., 2019).

The wider literature on anorexia indeed presents a discouraging picture. For example, the meta-analysis by Linardon et al. (2018) reported a dropout rate for eating disorder (ED) treatments of 24%, with some peaks reaching up to 70%. More recent studies (Marzola et al., 2021, 2022) have shown that about 40% of individuals undergoing treatment for an ED do not complete the full therapeutic process. This percentage rises to 50% for individuals with anorexia in inpatient treatment. In addition, around 20% of the completers become chronically ill. This is a result of the so-called “revolving door,” i.e., frequent relapses after hospitalization, leading to repeated admissions. Finally, anorexia mortality rates still hover around 10% (Khalsa et al., 2017) and, in many countries, it remains the disorder with the highest mortality rate among young people (Solmi et al., 2024; Touyz et al., 2023).

These results primarily concern inpatient and outpatient programs that largely follow a biomedical model (Toppino et al., 2024), which, in recent decades, has marginalized individual and family psychotherapeutic treatments by making them ancillary to medical-rehabilitation programmes. In this perpective, eating disorders are constructed as organic diseases, and the treatment has to focus only on symptoms and weight recovery.

## 2 Are the family base treatments an alternative to the prevailing medical model?

Family Base Treatments (FBTs) are, definitely, an alternative to the prevailing bio-medical model that denies the psychological and relational essence of eating disorders. The most empirically supported FBT (Baudinet et al., 2022; Eisler et al., 2016) –the Maudsley model-involves the patient’s family as a resource and tries to maximize the therapeutic alliance, especially with the parents. Its results are better than those obtained with the bio-medical programmes. However, they are not fully satisfactory. Certainly, the Maudsley model can reduce hospitalizations, associated with high relapse rates and subsequent readmissions (Eisler et al., 2022; Bentz et al., 2021). A remarkable result, no doubt. But the percentage of patients achieving good outcomes within one year of FBT ranges between 28 and 50% (Agras et al., 2014; Couturier et al., 2013; Lock and Le Grange, 2019; Madden et al., 2015; Datta et al., 2023). These unexciting results are at least partly, in our opinion, due to the prevailing zeitgaist that medicalizes the treatment of eating disorders from which even the FBTs have not completely escaped.

Although the Maudsley model draws on systemic conceptualizations and tools from the Milan strategic model (Selvini-Palazzoli, 1978; Selvini-Palazzoli and Viaro, 1988), the structural model (Minuchin et al., 1978), and the narrative model (White and Epston, 1990), it also contradicts some of their very core principles. First and foremost is the strong medicalization of the patient from the very onset of the disorder. This is a choice we do not agree with, just as we disagree with the much-touted agnosticism regarding the etiopathogenesis of anorexia and bulimia. Likely adopted to avoid the risk of blaming the family, this stance strips the disorder of its meaning and deprives the patient of agency, reducing her to a mere victim of an illness—akin to diabetes. Moreover, it undermines the very foundations upon which family therapy and its intervention methods are built. What is the point of family therapy if family dynamics play no role in the development of eating disorders? Deprived of many tools, the therapist, in our view, relies too heavily on symptom externalization. Is this White and Epston’s (1990) brilliant intervention, often effective in addressing many child symptoms, including encopresis, appropriate, especially for anorexia? We doubt. It certainly challenges the patients’ agency these adolescent and young women believe they can control their disorder, yet ultimately, they become its victims. But it dangerously aligns with the rhetoric patients with anorexia use to describe their refusal of food as an overpowering force they cannot resist. Symptom externalization, therefore, lends itself to being used by the patients, as it provides them with a medically supported justification to silence parents, friends, and relatives who accuse them of not wanting to eat. Even Maudsley therapists themselves have expressed concerns about the extensive use of this intervention (Aradas et al., 2019; Astrachan-Fletcher et al., 2018; Eisler et al., 2016; Lonergan et al., 2022).

## 3 The planned alternated pathways in and out of the systemic traditional approach on eating disorders

We believe, therefore, that there is ample room to explore alternative approaches, such as the planned alternating pathways we present here as a flexible protocol. We wish to emphasize its flexibility—psychotherapy cannot be confined to overly rigid guidelines. Families—including those with eating disorders—are diverse, as are anorexic patients; moreover, they both change over time. The therapeutic relationship itself also varies significantly. The therapist should therefore tailor their intervention to the specific case and the present moment.

Originally developed over the last twenty years by Ugazio (2010, 2019) and Ugazio and Fellin (2022, 2025) to address adolescent and young adult anorexia and bulimia, the planned alternating pathways are based on the theory of Family Semantic Polarities (Ugazio, 1998/2012/2018, 2013). This theory posits that in families where eating disorders emerge, conversation often revolves around a semantic of power, where the members who succeed, who can make their voices heard are seen as ‘winners’, whereas those who surrender are perceived as ‘losers’.

As well as the “winner/loser”polarity, these families have a second polarity – “strong-willed-yielding”—which is hierarchically dependent on the first, based on a relation of means to an end. These people are winners because they are willful, determined, efficient, or they are losers because they are passive, compliant, or liable to give in to others. Affability, amenability, acceptance of the definition given by the other person to the relationship are constructed within these families as passivity, faint heartedness, ineptitude (Ugazio, 2013, 182).

Unlike all other polarities, “the ‘winner/loser’ polarities cannot be perceived, even during the course of immediate experiencing, in terms of an individual trait, it relates exclusively to the relationship. It is the result of a comparison” (ibidem). One can only see themselves as a winner or a loser in relation to others. This peculiarity ensures that all family members maintain a constant and selective focus on others and their judgment. Hence, the struggle for relational definition and supremacy often becomes central, making the family context stingy with validation.

Members of these families, like all human beings, each want to be different. But the process of externalizing individual characteristics is obstructed. Since every self-definition is felt in comparative terms and produce feeling of superiority or inferiority towards others, the differences are immediately understood, but feared, denied, rejected and often considered unjustified (ibidem, 186).

The systemic protocol we are about to present differentiates itself from the classical model of family therapy. But it still remains faithful to some fundamental principles of the strategic model of Selvini-Palazzoli (1978) and Selvini-Palazzoli and Viaro (1988) and the structural model (Minuchin et al., 1978; Fishman, 2004) which continue to inspire certain lines of research to this day [see, for example, the program by Balottin et al. (2018), Cerniglia et al. (2017), and Mensi et al. (2020) on Lausanne Trilogue Play with families with EDs] and clinical practice in many countries. By re-establishing the link between family dynamics and eating disorders, our protocol, takes into account that the actors involved—the families, the eating disorders, and even the therapists themselves—are no longer the same as they were in the days when Minuchin and Mara Selvini Palazzoli built their pioneering models.

## 4 A tailored psychotherapy at the centre of the protocol

This protocol places a tailored psychotherapy at the center of treatment. Any necessary medical check-ups and diets are carried out autonomously by the patient and her family. In high-risk cases, the therapeutic team interacts with other health professionals, but always in the presence of at least the patient. The protocol adopts the pioneering view of Bruch (1973), Selvini Palazzoli (1963), and Minuchin et al. (1978) that anorexia, despite its risks, functions as a coping strategy. It is a sort of punch on the table with which the anorexic asserts the boundaries of her self and builds an initial nucleus of an autonomous identity. As Bruch (1962) already stated, anorexics suffer because “they act only in response to demands coming from others; they never feel that they do things because they want to” (p. 254). Their refusal of food is a way to break free from the “paralyzing sense of impotence” (ibid.), to assert one’s right to decide. This is a point with which Selvini Palazzoli (1963) fully agreed. For her too, emaciation is “a symptomatic expression of the search for security and power,” “a demonstration of the only possible autonomy for these patients. ‘They can force me to do whatever they want,’ said Azzurra (one of her patients), alluding to her parents, ‘but they will not be able to make me swallow even one more bite’” (Selvini Palazzoli, 1963, pp.103–104). This is an assumption on which many therapists and researchers still agree today. As Monteleone and Cascino (2021) affirm, “*Ineffectiveness, interoceptive ability and affective problems may be included in the core ED psychopathology, in addition to ED-specific symptoms*.”

## 5 The goals of planned alternating pathways

The protocol’s primary aim is to provide new relational positions for both the patient and her family. Crucially, it seeks to confer the patient a sense of efficacy that surpasses what anorexia can offer. Its objectives can be summarized as follows:

- Attacking the Disorder. Anorexia alters all significant relationships. The therapeutic objective is therefore to eliminate it or reduce its impact as soon as possible. Individual changes in the patient and in the family dynamic that do not lead to a symptomatic resolution are of little significance. However, according to us, it is better not to address the symptom directly.
- Focusing on relationships. Our therapeutic approach shifts the conversation from the food to the dynamics of the family and other vital contexts that sustain and motivate anorexia, deconstructing these dynamics and thus avoiding power struggles with the patient and coercive interventions.
- Deconstructing anorexic identity. This is achieved by avoiding, as much as possible, hospitalizations and interventions explicitly centered on weight and diet (food diaries, food-related tasks, etc.). The aim is to create a communicative space—the therapeutic space—where reframings, relational discoveries, along with the new positions experienced (and recounted) counteract the focus on food and diets. So, the attention and curiosity of the patient and other family members is redirected toward the issues at the root of conflicts and the disorder. The construction of the anorexic identity is not only a consequence of repeated hospitalizations; it is also the result of conversations dominated by weight and food.

These first three objectives belong to the systemic tradition, while the following are characteristic of this protocol and inspired by the theory of Family Semantic Polarities (Ugazio, 1998/2012/2018, 2013).

- Maximizing the therapeutic alliance. The predominance of the semantic of power in families where eating disorders develop renders the asymmetry of the therapeutic relationship particularly unpalatable for the members. Consequently, the therapeutic alliance is fragile (Lev Ari et al., 2023; Werz et al., 2021). Unlike Maudsley FBT, we intend to achieve this objective by adhering to a fundamental concept of the systemic model: the de-patientification of the symptomatic family member.
- Lengthening the treatment to address the emotional and identity issues of adolescent and young anorexics, which emerge forcefully when the disorder is overcome. Not coincidentally, anorexic patients, when they begin to regain weight, often experience a phase of deep sadness or depression, even during therapy. This decline in mood is attributable not only to the loss of the pathological power that the anorexic patient exerted over the entire family but also to the reemergence, in all its dramatic intensity, of the emotional and identity issues that the battle against food once contained. Addressing these issues requires a thorough therapeutic work with adequate timeframes. As we shall see, it is very difficult to expand the therapeutic process as much as necessary, but this objective can be achieved at least in part.
- Overcoming the typical therapeutic dilemma of eating disorders, which we summarize as follows: the psychotherapeutic process should be sufficiently long to address the identity issues of the anorexic or bulimic patient. However, the fragility of the therapeutic alliance necessitates brief treatments. As soon as therapy yields positive results and the patient regains a normal or near-normal weight, parents tend to abandon the therapy or place the therapeutic team in a position to terminate it. This is a phenomenon we have often observed in our clinical experience, and it has been confirmed by research data showing that stronger the therapeutic relationship is, the more severe the disorder, and vice versa (Lotempio et al., 2013).

The model we present also seeks to take into account the recurring difficulty of involving siblings, determined by the competitive dynamics fueled by the prevalence of the semantic of power in the conversations within these families. Often, the siblings are either reluctant to participate, present themselves as patients even in the absence of any symptoms, or their involvement is strongly opposed by the patient.

## 6 The planed alternating pathways: the protocol and its phases

The planned alternating pathways (Ugazio, 2010, 2019; Ugazio and Fellin, 2022, 2025) is designed to address the difficulties and dilemmas discussed earlier. The presence of a phase involving only the patient—previously anticipated to the entire family at the beginning of treatment—distinguishes this protocol from traditional family therapy while maintaining the same setting (one-way mirror, video recording, a team of therapists with one conducting the session). The goal of this individual phase is to allow an extension of therapy with the patient, who typically presents significant identity-related issues, in addition to the eating disorder.

As is well known, it is usually the parents who request therapy, often pressuring the anorexic or bulimic patient who is initially reluctant to participate. However, the therapist may become a valuable ally in the patient’s eyes if they gain the family’s (often ambivalent) trust during the family consultation and its extension. The protocol thus leverages the ability to form alliances—one of the typical resources of individuals within families dominated by the semantic of power—at a stage in therapy where this resource can be particularly useful.

Another characteristic feature of this protocol is the participation of the entire family in the final phase, reinforcing that the therapeutic work with the patient is rooted in the family dynamics already explored together.

In families that the parents have been separated or divorced for years and where at least one partner has established a new stable household, all phases involving family participation are conducted separately with the two family units. In cases of recent separations or divorces, the initial consultation is often conducted jointly, while the decision to continue consultation jointly or separately is carefully evaluated.

When there is open and intense conflict between parents, and the patient is clearly caught in the middle, the individual phase with the patient may be replaced or preceded by a phase involving only the parental couple.

### 6.1 Phase I: family therapy or individual therapy?

This phase, generally consisting of two sessions, each lasting two hours, is preceded by a phone call from the family member requesting therapy. A therapist from the team (Di Blasio et al., 1986) takes the call and agrees that the initial consultation will involve the entire family.

The first session begins with an in-depth discussion of the information already taken from the phone call, allowing the family to share their personal and family history with the therapist. This form of joining in serves to demedicalize the therapeutic context, engage all participants equally in the conversation, and implicitly reaffirm what the presence of all family members suggests: the patient’s disorder should be contextualized within the network of family relationships.

Because this introduction is so crucial, any attempts by the family to immediately introduce the problem are deliberately postponed. The transition to exploring the disorder is marked by therapist with statements such as: “Great. Now that we know each other, we can dive into the issue.” Then the therapist maps the eating disorder and its pragmatic effects within both the nuclear and extended family with question such as: “When did the eating disorder first emerge? How did it develop? Who noticed it first? Who is aware of the problem and the request for therapy? What has changed since the patient developed anorexia?”

The goal is to obtain as clear a picture as possible of the family organization *before* and *after* symptom onset and the related differences. The first session concludes with an exploration of the explanations that various family members have given about why the patient developed anorexia, along with what events they believe contributed to its onset.

The second session focuses primarily on analyzing the “politics” the family has organized around the eating disorder (Sluzki, 1992), following the classic systemic method. This includes examining attempted solutions, including previous therapies and their effects. Special attention is given to the pattern through which the family has connected to psychotherapy, the underlying family dynamics, and each family member’s expectations regarding the therapeutic process (Ugazio, 1989). At the end of this session, the therapist recommends that the family extend the consultation phase for a limited number of sessions (ten to sixteen at most) providing the following justifications:

- family events related to the onset of the disorder need further exploration;
- an occurrence almost always present: the patient, who had previously been engaged in external activities as expected for her stage of life cycle, has withdrawn into the family unit, now her only point of reference.

The therapist also mentions that there will be a phase focusing solely on the patient. Announcing this phase helps prevent escalating conflicts between those who prefer family therapy (usually the patient and the parent who sees themselves in a “losing” position) and those who favor individual therapy. All perspectives are hence welcomed, but only partially.

### 6.2 Phase II: everyone must win, everyone must be rewarded…

The primary goal of this phase, which is more directly aimed at change and involves the entire family, is to empower the family as a whole, while mitigating internal competition and the polarization between “winners” and “losers.” The position within the semantic of power should be redefined, particularly by ensuring that members in a losing position are empowered (Ugazio, 2019). As in Dodo’s verdict, everyone must win and deserves a prize.

The point is not to liberate the family from the semantic of power by introducing meanings foreign to its history. The very semantic of power is broad enough to allow for the creation of new and different polarities and narratives that are not linked to, or exacerbated by, the disorder. These new narratives are also more readily accepted by the family, because they align with their emotions, values, perceptions, and ways of positioning with each other. What therapy should counteract, however, are the pathogenic assumptions that can be summarized as follows:

- Power as a limited resource: Bateson (1979) argued that it is not power that corrupts, but rather the idea that one part of the system can unidirectionally dominate the other. This notion is certainly present in these families—after all, the anorexic girl believes she can control her body through sheer willpower. This belief is often accompanied by another, equally dangerous one: that power is a fixed and limited quantity that can be taken away. If I acknowledge that you are beautiful, intelligent, and competent, am I ugly and incapable? This reification of power is divisive, as it hinders cooperation and fuels destructive competitions.
- Competitive trails: In these families, everyone tends to compete with and emulate the “winners” (or supposed winners) in their field or to become discouraged if they lack the necessary resources. Even against their will, “winners” end up acting as Pied Pipers, leading their competitors to follow their trail, ignoring their *own* talents, skills, and inclinations in favor of *those* that guarantee success to them. For example, no one might notice that the daughter excels at drawing because the family’s competition revolves around music. Everyone evaluates and compares themselves based on their musical abilities, and many pursue music professionally—often suffering—because no one can reach the heights of the uncle, a celebrated orchestra conductor.
- Devaluation of feelings and emotions: When the semantic of power prevails, emotionality and empathy are seen as dangerous—if you are too in tune with your family members, you inevitably fall victim to their need to dominate. The “losers” in the family are often those who are more emotional and empathetic than others. Their resentment, which plays a significant role in instigating processes that target the patient (Selvini-Palazzoli, 1978; Ugazio, 1998/2012/2018, 2013), stems mainly from the lack of recognition and devaluation of their often essential emotional role within the family.
- Equality as the suppression of differences: This assumption underpins the semantic of power and is responsible for the suspicion with which differences are viewed, because they are framed in terms of superiority and inferiority (Ugazio, 1998/2012/2018, 2013). Within this semantic, acknowledging a difference means opening oneself to the possibility of humiliation. As a result, although everyone in the family aspires to have their uniqueness recognized, they are unable to differentiate themselves. Conformity thus becomes dominant, reinforcing competitive trails.

Since these trails and assumptions are deeply embedded in our culture, they often shape the mindset of therapists as well. It is therefore essential for the therapeutic team to free themselves from these biases through reflective practices, in order to address the primary task of this phase: redefining meanings and roles within the family to ensure that every member holds a valuable position and takes pride in their contributions to the family unit.

These transformations of meanings, which ensure all family members an honourable position, are achieved also through reframing, micro-reframing, and falsifying experiences (Ugazio and Ferrario, 1992) and always tailored to the specific family. It has a common objective: to validate emotions and feelings, recognizing their centrality in changing the perception that so-called “losers” have of themselves, and that other family members have of them. “Winners” and “losers” are redefined, respectively, as “task-oriented” and “relational-oriented” individuals, both essential to the family unit precisely because of their distinct competencies. Any other polarities that create devalued, despised, or pitied positions should also be transformed during this phase.

The interview technique introduces a new protagonist: the nuclear family as a group. Competition often fragments the boundaries of the nuclear family, leading parents to identify more strongly with their own family of origin, and children to align themselves with one side of the extended family over the other. Through targeted questions that compare the nuclear family with extended family networks, friends, and social institutions, the therapist reconstructs these boundaries, reinforcing the identity of the nuclear family unit and securing a dignified position for it in relation to other groups. The construction of this new subject naturally reduces competition, as the supposed “winners” now contribute to a collective advantage, making cooperation within the family an asset.

### 6.3 Phase II: the transition to individual therapy

The transition to individual therapy for the patient, which marks the end of this phase, is a delicate step. Even when the eating disorder has been overcome or is nearly resolved, this transition can be interpreted as confirmation that the other family members have little or nothing to do with the patient’s problems, which she should now face alone. It is therefore crucial that the individual therapy phase is not presented as a treatment for the patient’s “illness” or, worse, her being deficient, inferior, or the “broken piece” of the family. If perceived in this way, the treatment would be dangerous.

Thus, the second phase concludes with a reframing that justifies the transition to individual therapy based on the family dynamics that have emerged during the consultation. The family is also informed in advance that therapy will end with a joint phase.

During the individual phase, parents and siblings can call the therapeutic team to provide information about events or situations concerning the patient or other family members. They may also request a family session if they deem it necessary. The therapists will not provide them with any information, but will inform the patient of what has been communicated and discuss with her whether to hold a family session. Additionally, the therapists, in agreement with the patient, may request one or more sessions with the entire family, part of the family, or a single member to clarify situations, perceptions, or problems that are difficult to understand.

### 6.4 Phase III: the individual path

The transition to individual therapy usually occurs when the patient has already achieved significant weight recovery or even reached a normal weight. If this transition is motivated by the family dynamics explored together, it is generally well accepted by the patient, sometimes even with enthusiasm. Like other family members, the patient is skilled at building alliances and using them strategically. Hence, she often sees the therapist as a powerful ally—unfortunately in opposition to her parents. Of course, the therapist redirects this alliance toward the patient’s emancipation, primarily from the eating disorder and to foster changes in the overall family dynamics.

In any individual therapy, systemic therapists consider the patient’s family members as virtual actors in the therapeutic process, as they can influence the therapy’s course and, in turn, being influenced by it. This is particularly true for this phase of the protocol, which remains part of the family therapy journey. Therapists should therefore be particularly attentive to the family dynamics that develop around the individual path.

We strongly suggest that emotions, feelings, moods are left out of the therapeutic conversation, especially at the beginning of the individual phase. If the therapists were to explore this area where the patient feels most incompetent too soon, the patient’s feelings of discomfort and inadequacy would intensify. Instead, the therapeutic work will focus—ideally in sequence but sometimes simultaneously—on the four areas outlined below:

1. *The patient’s relative positioning:* As in the previous phase, attention remains on family relationships and the positioning of each family members. Now the focus is on how each nuclear and extended family member positions themselves toward the patient and vice versa.
2. *the pragmatic and identity effects of the patient’s and siblings’ positioning on the parental couple, and vice versa:* When the patient is not an only child, significant attention is given to exploring how siblings position themselves in relation to the parental couple and vice versa. This serves two main purposes. First, it allows the patient to autonomously identify her own position and its changes within the family by comparing herself to her siblings. Second, it facilitates the creation of a generational bond among siblings, usually present in adolescence but often difficult to establish in these families due to intense competition and frequent intergenerational alliances and coalitions.
3. *understanding others’ emotions:* By deciphering the emotions and seemingly enigmatic behaviors of family members and intuiting their motivations, the patient inevitably begins to shape her own internal states, enhancing her sense of personal competence.
4. *discovering hidden talents suppressed by “competitive trails”:* What does the patient excel at effortlessly? What activities bring her joy or satisfaction? These questions, while not directly focused on emotions, inevitably involves them. Exploring them, the therapist often highlights patient’s abilities demonstrated during sessions or subtly emerged in the patient’s and the family’s narratives. The therapist employs selective attention to unrecognized strengths, which have been expressed throughout the entire therapeutic journey. The goal now is to help the patient focus on her own talents. This work, usually conducted toward the end of the individual phase, often aligns with educational or career choices—decisions that, once the eating disorder has been overcome, become urgent and unavoidable in the patient’s life cycle.

This individual phase strengthens the patient-therapist alliance, with the significant advantage of paving the way for the patient’s possible return to therapy, should existential issues arise in the future, particularly those related to the identity fragility previously discussed. This is an important outcome, as this phase of therapy is almost always brief. Parents often seek to shorten this phase, especially once the eating disorder has been overcome. In our clinical experience, very few individual phases have lasted more than twenty sessions, and it has generally been the parents who determined the conclusion. The patient herself can also contribute to prematurely ending this phase, as she tends—sometimes subtly, sometimes overtly—to position the therapist in opposition to her parents. The decision of several patients in our clinical sample to pursue a university degree in psychology is emblematic of this dynamic. It is a particularly painful move against parents who, for years, have presented themselves to their children as role models.

It is also worth noting that while we allow for the possibility of meetings with other individual family members, we rarely resort to them. Instead, when behaviors or events arise that are difficult to interpret, we prefer to encourage the patient to initiate conversations with family members to gather missing information. This approach restores agency to the patient and prevents the triggering of family dynamics that might be challenging to manage.

### 6.5 Phase IV: a joint conclusion

The joint conclusion with the whole family, announced at the end of the second phase, reaffirms the family framework of the therapeutic journey, therefore it is essential. All the changes the family has achieved or initiated are explored during this phase. As the systemic model teaches us, if the patient has overcome the disorder and truly modified her positioning, other family members will also have undergone significant changes—sometimes even transformative shifts. Naturally, this exploration also includes an assessment of the eating disorder. Has it been completely resolved? What still needs to be addressed? Equally detailed is the analysis of changes in other family members and subsystems—both nuclear and extended—directly or indirectly connected to the problem.

If serious issues persist or emerge, we negotiate an extension of the therapy. If previously unspoken marital problems are mentioned during this final phase, or difficulties among siblings become apparent, we suggest addressing them with other colleagues. When the eating disorder seems resolved and we observe a substantial improvement in family conversation, we declare the therapy completed and schedule a follow-up, usually one year later.

It should be noted that the family may decline the invitation to this concluding phase—sometimes one or two members may claim they are unable to attend. This situation is not uncommon when the patient is doing particularly well. In such cases, we make every effort to ensure the participation of all reluctant or unavailable members. If they cannot attend in person, we involve them online. We then hold a session with the patient, the family members present in person, and those connected remotely. If it is not possible to have everyone together, whether in person or virtually, we arrange multiple sessions, ensuring that the patient is always present while other family members join remotely. The key is that everyone takes part in this phase.

The participation of all members in the follow-up session or sessions —typically conducted a year after therapy concludes—is also pursued with the utmost commitment by the team. The follow-up is an integral part of this final phase of the protocol. During this session, we initiate a dialogue with all family members about their experience with therapy, including the individual phase with their daughter. How did they feel with the therapists? Did they feel welcomed? Supported? Understood? Were there difficult moments? Did they ever consider abandoning the treatment? Would they go through the process again? We conclude this evaluation by asking for suggestions: is there anything we could change, avoid, or introduce to improve the therapeutic process?

The dedication and consistency with which the therapeutic team strives to ensure everyone’s presence at the final phase reinforce—on an implicit, yet deeply significant level—that every family member is involved in the problem, without exception. The unspoken message conveyed is that anorexia or bulimia —by this point usually overcome—was an issue that affected the entire family.

## 7 Conclusion

The protocol presents a novel therapeutic strategy for working with families with eating disorders, where the prevalence of the semantic of power generally hinders the construction and maintenance of a strong and enduring therapeutic alliance. This semantic also jeopardise the compliance and completion of the interventions and hence their positive outcome. Indeed, the typical therapeutic dilemma of eating disorders entails that therapy should last long enough to address the core identity issues of the anorexic or bulimic patient, but the perceived asymmetry of power causes often premature drop-outs. Often, as soon as the patient restores a normal or near-normal weight or achieves a significant symptom reduction, the parents urge the end of the therapy or put the therapeutic team in the position of terminating it. The introduction of an individual phase helps to prevent the too early conclusion of therapy. Only after the empowerment of the family as a whole, and of the patient’s self-esteem, the individual path can carefully approach the patient’s emotions, feelings and moods. This is achieved in an indirect way, while helping the patient to discover her hidden talents and passions, often suppressed by the dominant “competitive trails.” This individual phase also paves the way for a possible return to therapy, should existential issues arise in the future, as we have often observed.

The protocol presented and discussed here has so far been developed and applied with adolescents and young adults with anorexia and bulimia and their families. Most of our cases, consistently with the prevalence reported in the literature, were girls or young women. However, the proposed intervention protocol could be extended to work with other client groups, of any gender, and could be adapted also for other EDs. It can also be extended to other treatments for adolescents or young adults facing other issues and disorders, especially those dominated by the semantic of power. For instance, we successfully applied it to families with suicidal self harming (SSH) and not suicidal self harming (NSSH) adolescents. These directions are indeed envisaged in our future investigations.

The format that distinguishes this approach takes into account the changes that have occurred in all families—regardless of the disorder—that make it difficult for the whole family to participate in rather lengthy therapeutic paths, especially in the life cycle stage when the children are adolescents or young adults. The academic and professional commitments of siblings and the additional complexities of blended families are among the factors hindering the long-term involvement of the whole family in the therapeutic experience.

Additionally, the protocol reduces the asymmetry between patients and therapists while enhancing the family’s resources. Intervention models such as the strategic approach of Selvini-Palazzoli (1978) and Selvini-Palazzoli and Viaro (1988) or structural models (Minuchin et al., 1978) appear unsuitable today. Western societies, increasingly resistant to hierarchical relationships, are uncomfortable with prescriptive therapeutic models. This protocol may not be so useful for therapists working with families less affected by power dynamics and with patients from non-Western backgrounds, where family hierarchies, conceptions of “power,” and adolescent autonomy could differ significantly. However, this is merely a speculation, because we have no empirical evidence on this point.

Despite some significant strengths, this protocol suffers from some strong limitations as well. The absence of adequate empirical support through the application of the protocol to the treatment of a substantial number of adolescents and their families with an eating disorder of similar severity and nature is the most important limitation. So far, we have applied the Planned Alternating Pathways to only 21 families with patients with adolescent anorexia and bulimia. Some of our patients, particularly bulimic ones, presented comorbidities, such as SSH or NSSH, severe substance abuse (e.g., ethyl coma) and obsessive-compulsive disorder. Eighteen of the 21 treatments were successful, with achievement of a normal weight and significant positive changes in the social life of the patient and family members at the end of therapy or at follow-up at one or two years after the end of therapy. In four cases, however, the treatment ended at the extension of the consultation. The patient had reached a normal weight and the family felt that the therapy could end without moving on to the individual phase. There were two drop-outs. In one case of anorexia with SSH, the family wanted to move to the individual phase after the initial consultation, a request we did not accept. In the second case, the treatment was interrupted during the extension of the consultation because the patient, a male adolescent with severe anorexia with comorbidity with obsessive-compulsive disorder, had worsened after an initial improvement. In addition to these two drop-outs, we discontinued a treatment on our own initiative of a case of anorexia, to the disappointment of the parents, because of a mismatch of therapeutic goals with hospital teams, who were following the case with us, who chose to intervene pharmacologically and with a hospitalisation, which in our opinion was not necessary.

The limited number of cases whose treatment has been conducted according with this protocol, and especially their stark differences in terms of severity and presence/absence of comorbidity, clearly jeopardizes data generalizability. Hence more extensive practice-based research is needed, whereas evidence-based methods such as randomized controlled trials (RCTs) are incompatible with the tailored and flexible approach here presented.

In the following article, we will illustrate in detail the application of our protocol to a clinical case of adolescent anorexia.

## Data Availability

The original contributions presented in the study are included in the article/supplementary material, further inquiries can be directed to the corresponding author.
